# Parental Factors Moderate the Association Between COVID-19 Disruption and Adolescent Emotional and Behavioural Difficulties

**DOI:** 10.1177/02724316241311131

**Published:** 2024-12-23

**Authors:** Kate Van Kessel, Charlotte Aitken, Elizabeth S. Nilsen

**Affiliations:** 1 8430 University of Waterloo, Canada

**Keywords:** adolescence, emotional and behavioural difficulties, parental reflective functioning, parental support, COVID-19, parental stress

## Abstract

Past work shows that COVID-19 impacted adolescent mental health, but the moderating role of parental factors remains unclear. Ninety-one parent-adolescent dyads (ages 12–15) completed online surveys. Parents reported on COVID-19 disruption within their household, their mental health, parental reflective functioning (i.e., ability to consider the mental state of their child), and their adolescent’s emotional and behavioural difficulties. Adolescents rated their own emotional and behavioural difficulties and perception of parental support. Positive associations between household COVID-19 disruption and adolescent difficulties emerged, regardless of informant. However, parental factors linked to adolescent difficulties varied by informant. Parental reflective functioning moderated the association between COVID-19 disruption and adolescent difficulties (parent-report). COVID-19 disruption showed some stronger associations with adolescent difficulties than other parental stress measures, but not consistently. Findings replicate and extend prior work, emphasizing the negative association between COVID-19 disruption and adolescent mental health, while highlighting parental reflective functioning’s potential for mitigating adolescent difficulties.

## Introduction

As the long-term effects of the COVID-19 pandemic emerge, studies have documented the negative impact on the mental health of parents and children, noting symptoms of depression, anxiety, and loneliness beyond pre-pandemic trajectories (De France et al., 2022; Ma et al., 2021; Westrupp et al., 2021). The magnitude of the effect of COVID-19 on well-being has been shown to vary across different age groups, with higher rates of mental health problems associated with adolescents as compared to pre-school children and older age groups (Cost et al., 2022; Ma et al., 2021; Varma et al., 2021). However, there was much variability in adolescents’ responses to the pandemic (Cost et al., 2022; Coulombe & Yates, 2022; Waite et al., 2021), emphasizing a need to understand the factors that contribute to adolescent outcomes during times of societal disruption, such as COVID-19. One factor that may be important to consider is the role of parents, who act as a relational influence throughout this developmental period.

The primary aim of the present work was to explore how parental factors may be associated with adolescent emotional and behavioural difficulties (hereon referred to as adolescent difficulties for brevity) within the context of the COVID-19 pandemic (with the majority of data reflecting the experiences of Canadian families). Specifically, we examined how parents’ experience of COVID-19 disruption within the household was associated with adolescents’ difficulties and whether parental factors moderated the association between COVID-19 disruption and adolescent difficulties. Understanding the role of parents in supporting adolescents may be especially important, as previous research suggests that parents play a significant role in promoting adolescents’ resilience through times of stress (Cheraghian et al., 2023; Gagné, 2003; Masten, 2014; Zimmer-Gembeck & Skinner, 2011). However, environmental and societal disruptions impact all members of a family and may present unique barriers to parental support. Further, while some of the underlying mechanisms of parental stress and adolescent functioning may be similar across different types of stressors, COVID-19 disruptions introduced unique challenges (e.g., widespread and unpredictable disruptions to daily life). Thus, a secondary aim of the present work was to examine whether patterns of association with COVID-19 disruption to a household are distinct from other parental stress measures.

Understanding the role parental factors play for adolescent functioning during COVID-19 provides key insight into how best to support youth and families as they navigate unpredictable circumstances, which is particularly important as we continue to see a rise in wide-reaching societal disruptions (e.g., increased likelihood of future pandemics, rising incidences of natural disasters that may require evacuation or disrupt schooling, war/civil unrest).

### COVID-19 Disruption and Adolescent Well-Being

Adolescence is a critical period of social-emotional vulnerability characterized by changes associated with puberty, cognitive development, and evolving relationship roles, all of which can negatively affect mental health and increase problem behaviours (Casey et al., 2008; Gutman & Eccles, 2007; Paus et al., 2008). Further, adolescence is marked by an increasing interest in developing independent identity, fostering autonomy, and developing peer relationships (e.g., Brown & Larson, 2009; Greenberg et al., 1983; Oudekerk et al., 2015; Scholte & Van Aken, 2007; Zimmer-Gembeck & Collins, 2006), all of which were highly impacted by COVID-19 restrictions (e.g., Nadeem & Van Meter, 2023). With the onset of the COVID-19 pandemic, adolescents’ routines were disrupted with public health measures mandating the closure of businesses, schools, and recreation activities during the stay-at-home period. These public health measures restricted adolescents’ ability to engage in social activities and led to the implementation of remote learning. During the onset of the pandemic in Canada, adolescents reported being concerned about family finances, infection, schooling, friendships, and their reputations (Ellis et al., 2020). Trend analysis of child and adolescent samples showed evidence of an increase in anxiety and depression symptoms during the COVID-19 pandemic compared to pre-pandemic estimates, especially among female individuals (Madigan et al., 2023). Systematic reviews reiterate the findings of increased levels of anxiety, depression, worry, loneliness, sedentary behaviours, social media use, and behavioural problems among adolescents during the pandemic (Elharake et al., 2023; Kauhanen et al., 2023; Mucci et al., 2024; Scapaticci et al., 2022). A longitudinal study conducted in Iceland with adolescents found that the increase in depressive symptoms and the decrease in mental health well-being among 13–18-year-olds persisted 1–2 years into the pandemic (Thorisdottir et al., 2023). Though, person fixed-effects models assessing changes in adolescents’ mental health during the pandemic found that the pandemic’s impact on youth mental health varied based on their pre-pandemic mental health (Hu & Qian, 2021). Given these significant disruptions, it is important to understand how other factors can mitigate potential negative mental health effects for adolescents during disruption.

Within the context of the COVID-19 pandemic, one way in which the social and environmental changes may particularly impact adolescent well-being is by influencing the family context (Araújo et al., 2020). With the restrictions on adolescents’ peer relationship and increased time at home during this time, the influence of parents on adolescent well-being (Greenberg et al., 1983; Helsen et al., 2000; Youniss & Smollar, 1985) may be especially significant. Moreover, lockdowns increased work-family conflict, parenting stress and irritability, and couple conflict (Chung et al., 2020; Low & Mounts, 2022; Operto et al., 2022; Prime et al., 2021; Roos et al., 2021). These factors lead to lower-quality parenting and more negative interactions between parents and adolescents, which in turn predicted poorer child mental health problems (Donker et al., 2021; Low & Mounts, 2022; McArthur et al., 2023; Operto et al., 2022; Peltz et al., 2023; Romero et al., 2020; Shelleby et al., 2022; Westrupp et al., 2021). Notably, researchers have identified both positive and negative effects of COVID-19 disruption on family functioning. For instance, a longitudinal study by Wang et al. (2021) demonstrated that parental employment status during the COVID-19 pandemic had varying effects on adolescent emotional well-being: parents working from home were more likely to display parental warmth – positively predicting adolescent emotional well-being; parents who reported job loss were more likely to report parent-adolescent conflict – negatively impacting adolescent emotional well-being.

### Potential Moderating Parental Factors

Theoretical models posit that adolescents are affected by parental factors, including during periods of disruption (Masarik & Conger, 2017). Parental behaviours influence the quality of the parent-adolescent relationship, which in turn predicts adolescent outcomes. A longitudinal study with more than 8000 participants established a significant link between specific modifiable aspects of parent-adolescent relationships, such as parental warmth, communication, and time spent together during adolescence, and favorable health behaviors and outcomes in young adulthood across various domains, including general health, mental health, sexual health, and substance use (Ford et al., 2023). Parent factors may be especially important for understanding adolescent outcomes during the COVID-19 pandemic given the limited interaction adolescents had with peers and other adults/mentors during this time, resulting in potentially higher influence of parents on their adolescent children. In the current study, we examine whether the parental factors of parental reflective functioning ability, parental depression, and adolescent’s perception of parental support impact the degree to which COVID-19 disruption is associated with adolescent’s well-being. These specific factors were chosen as past work demonstrates their association with adolescent difficulties (Benbassat & Priel, 2012; Sheeber et al., 1997; Thapar et al., 2012), as well as possible susceptibility to stressors (Donker et al., 2021; Nelson et al., 2020). For instance, adults who are under greater stress engage in more self-focused (vs. other-focused) mentalizing. Thus, it is possible that elevated COVID-19 disruption may place demands on parents’ abilities to reflect upon the mental states of their adolescent and provide supportive behaviours.

#### Parental Reflective Functioning

Parental reflective functioning (PRF) is defined as a parent (or caregiver’s) ability to consider themselves and their child as separate agents with their own beliefs, feelings, goals, and desires that guide behaviour (Fonagy et al., 1991; Fonagy & Target, 2002). PRF encompasses both a parent’s ability to think of their child as having their own mental states and a parent’s ability to reflect on how their behaviour may affect their child. Examples of strong PRF include accurately recognizing and validating adolescents’ emotions, expressing curiosity about the underlying intentions and feelings behind their child’s behaviour, accurately inferring their child’s intentions and goals, and demonstrating this attunement to their child through empathetic listening and/or responding to needs (e.g., offering reassurance during stressful times). Luyten et al. (2017a) conceptualized one form of ineffective PRF as *pre-mentalizing*, or parents’ repudiation of their child’s mental experience and/or maladaptive attributions about their child’s thoughts, feelings, and behaviours. Examples of ineffective PRF, or pre-mentalizing, include parents dismissing their child’s feeling, difficulties recognizing one’s child as having complex or nuanced internal states, misattributing their child’s intentions (e.g., accusing the adolescent of being lazy when they are actually overwhelmed or anxious), focusing on external behaviours without considering mental states, and non-responsiveness to needs (e.g., being emotionally unavailable, possibly due to an inability to recognize the child as a fully independent agent). Strong PRF has been associated with positive parental behaviours (Smaling et al., 2016), whereas weaker PRF is associated with negative parenting behaviours (e.g., difficulty tolerating or regulating their children’s negative emotions and lower distress tolerance; Kelly et al., 2005; Rutherford et al., 2015). Strong PRF also relates to child outcomes, with better parent reflective functioning being associated with children’s own regulation skills, including better child affect regulation, more secure emotional attachment, and better emotional understanding (Fonagy et al., 1991; Steele et al., 1999). However, markedly less research has examined the role of PRF within adolescence, despite the ongoing cognitive, emotional, and social changes that occur in this developmental period. For instance, adolescents continue to develop perspective-taking and mentalizing skills (Benbassat & Priel, 2012; Gabriel et al., 2021) and their overall development may be especially sensitive to social influences. These developmental changes that occur during adolescence may mean that the observed associations with PRF in childhood look different in adolescence. For example, Benbassat and Priel (2012) found that better PRF was associated with more internalizing problems amongst adolescents (14- to 18-years old), unlike findings from prior research with younger children (7- to 11-years old), where maternal RF was associated with less child psychopathology symptoms (Sharp et al., 2006). That said, Benbassat and Priel’s (2012) findings with adolescents is consistent with findings from postsecondary students wherein better mentalizing (albeit within the same individual) was associated with negative emotional states, such as greater self-criticism and anxiety (Farber, 1989), as well as depression (Gara et al., 2002). Benbassat and Priel theorize that being more adept at understanding mental states, including those of one’s child, may also lead to greater awareness of negative states, which could increase the risk for internalizing behaviours (though, it is important to note that the levels of internalizing remained within the normal (i.e., non-clinical) range). Benbassat and Priel (2012) also found that adolescents’ own reflective functioning moderated relationships between parent behaviours and adolescent adjustment, suggesting that there may be nuanced relationships between parenting skills, parental behaviours, and adolescent well-being.

A number of studies (Stob et al., 2020; Zayde et al., 2023) have found that through interventions directed at improving PRF, child and parent outcomes were improved – including some preliminary evidence in the context of COVID-19 (Zayde et al., 2023). Moreover, during COVID-19, the indirect effect of mother’s anxiety symptoms and preschool-age children’s externalizing behaviours was weaker among mothers who displayed better mentalization skills (Dollberg et al., 2021). Given the importance of PRF in buffering the indirect effect of parent anxiety on behavioural outcomes of younger children during the COVID-19 pandemic, PRF could play an important role in offsetting the impact of parent-experienced disruption on adolescents. Here we examine parental experience of COVID-19 disruption and adolescent difficulties to understand how PRF may relate to these variables. It may be that associations observed between better PRF and child outcomes (Slade, 2005; Smaling et al., 2016; Steele et al., 1999) carry into adolescence, namely, negative association between COVID-19 disruption and adolescent difficulties would be attenuated amongst parents with stronger PRF skills. However, as associations between PRF and adolescent outcomes have been demonstrated to deviate from patterns with children (see Benbassat & Priel, 2012), it is also plausible that we may find a stronger association between COVID-19 disruption and adolescent difficulties amongst parents with stronger PRF skills.

#### Parental Depression

Parental mental health is an important factor that influences the parent-child relationship and may have downstream effects on adolescents’ own mental health through a variety of pathways. For example, adolescents whose family members have a history of depression are at a high risk of developing mental health difficulties such as depression (Natsuaki et al., 2014; Silberg et al., 2010; Thapar et al., 2012). Wilson and Durbin (2010) proposed that parental depression affects the parent-child relationship via a lack of positive parenting practices and overuse of negative parenting practices (although see Shafer et al., 2017). During the pandemic researchers documented an increase in adult reports of depressive symptoms (Nelson et al., 2020; Olagoke et al., 2020; Pinchoff et al., 2021). The dysregulated prefrontal cortical functionality observed with depression may impair the ability of parents with depression to understand and respond appropriately to adolescents’ needs (Arnsten et al., 2021; Fischer-Kern et al., 2008; Hare & Duman, 2020). Thus, parental depression may undermine coping efforts during COVID-19 and thus relate to greater adolescent difficulties. Given that parental depression is posited to negatively affect the parent-adolescent relationship we predicted that parental depression would strengthen the association between COVID-19 disruption and adolescent difficulties.

#### Parental Support

Parent-child relationships are a main source of support during childhood and into adolescence. Positive and reliable engagement within the parent-child relationship empowers children to seek parental support when in need (Thompson et al., 2008). Parental support such as praising that indicates acceptance (Barnes et al., 2000) is a key factor facilitating adolescent’s transition to adulthood. Furthermore, adolescents’ perceptions of higher parental support have been linked to less internalizing and externalizing problems (Barnes et al., 2006; Gorostiaga et al., 2019; Sheeber et al., 1997; Wang et al., 2013). In the context of the COVID-19 pandemic, a longitudinal study by Donker and colleagues (2021) found a decrease in adolescent’s perceptions of parental support. Given the previous work identifying parental support as a protective factor for adolescent emotional and behavioural outcomes, we predicted that parental support would weaken the association between COVID-19 disruption and adolescent difficulties.

### Current Investigation

The current study examines the degree to which parental experience of household disruptions, caused by COVID-19, were associated with adolescents’ difficulties. As indicated in the sections above, our particular focus was how parental factors, namely, PRF, parental depression, and adolescent’s perception of parental support moderate such associations. Understanding the role of parental factors during this developmental stage provides key insights into how best to support adolescents and families as they navigate unpredictable circumstances. These insights extend beyond the context of the pandemic, offering a broader understanding of how parental factors can affect adolescent outcomes during periods of significant stress and transition.

Importantly, to minimize shared method variance, and account for the fact that adolescents and their caregivers may have different insights, both adolescents and parents reported on adolescents’ difficulties. Indeed, developmental researchers often use a multi-informant approach to measurement, as behaviour is known to vary significantly across situations, and informants hold unique perspectives that contribute to informant discrepancies (Booth et al., 2023). Studies demonstrate low to moderate parent-child agreement on reports of child behavioural and emotional problems, with greater discrepancies with reports from adolescents aged 12- to 19- years old as compared to children aged 6- to 11- years old, and for internalizing as opposed to externalizing problems (Booth et al., 2023; De Los Reyes et al., 2015; Sawyer et al., 1992; Sourander et al., 1999). Parents tend to report reduced youth emotional and behavioural problems as compared to adolescents themselves (Booth et al., 2023; Sawyer et al., 1992; Sourander et al., 1999), with the nature of discrepancies differing by adolescent sex and parental factors (Booth et al., 2023). Discrepancies arise from differences in what/how parents and adolescents use information to form their judgements reflecting the subjective perspective of that informant’s observations and situational experiences (e.g., observability of behaviours; Karver, 2006). Moreover, points of divergence in multi-informant reports have the potential to reflect situational specificity, namely differences in how adolescent’s emotional and behavioural difficulties manifest in distinct settings (e.g., home vs. school; De Los Reyes et al., 2022). Given the inherent situational specificity of the experience and expression of adolescent difficulties, discrepancies between parent and adolescent reports are common and provide unique, context-specific insights that capture a more holistic view of adolescent emotional and behavioural functioning. Thus, research using both self- and parent-report is needed to clarify how, or with what parental factors, disruption might relate to adolescent emotional and behavioural functioning.

In the immediate years following the onset of COVID-19 there has been a significant body of research examining the effects of COVID-19 disruption on families and a key challenge moving forward is understanding whether the findings with COVID-19 disruption are novel or if they can be generalized to other stressors. Given that very little work has examined whether patterns of association with COVID-19 disruption are distinct from other parental stress, we also investigated whether associations between COVID-19 disruption with adolescent difficulties would differ with non-COVID-specific measures of stress, including major life stressors and perceived stress. COVID-19 was a novel situation and as such, parents may have been ill-equipped to manage this type of disruption as opposed to other life stressors. If this was the case, then we may observe a greater association between COVID-19 disruption and adolescent difficulties as compared to major life stressors or perceived stress. Understanding whether COVID-19 disruption showed similar patterns of association affords a unique opportunity to examine the extent to which insights drawn from studying parents’ responses to COVID-19 disruption can be extended generally to how they respond to other types of stress.

## Method

### Participants

Parent-child dyads were recruited through a through a lab database at the University of Waterloo, advertising with local high schools and community organizations, and the online research platform Children Helping Science. In total, 98 adolescents aged 12- to 15-years-old and 109 caregivers participated, but only data from complete dyads (*N* = 91) were included in analyses. Participants were predominantly from Canada (86.81%), with a few from the United States (7.69 %) and other countries (e.g., Australia, 5.49%). Data was collected between April 2021 and March 2022.

#### Parents

Of the 91 parents, 77 (85%) reported they were female and 14 (15%) male. Parents ranged in age from 37- to 63- years old with a mean age of 45.80 years old (*SD* = 4.80). The majority of parents (90%) reported being very comfortable understanding written English and reported White/Caucasian as their ethnicity (79%). The second- and third- most reported ethnicity was East Asian (10%) and South Asian (9%), respectively. The remaining ethnicities reported were 2% Indigenous, 1% Latinx, 1% Middle Eastern, 1% Guyanese Indian, and 3% Biracial. The majority of participants reported that they held an undergraduate degree or higher (86%), as did the adolescent’s other parent (90%), with 97% reporting at least some post-secondary education. Most parents reported a household income of above $75,000 (82%), which is roughly the median income for Ontario, Canada (Statistics Canada, 2022) - the province from which the majority of our participants were recruited.

#### Adolescents

Of the 91 adolescents, 46 (51%) reported they were female, 44 (48%) male, and 1 genderfluid (1%). Adolescents ranged in age from 12- to 15- years old with a mean age of 13.37 years old (*SD* = 1.15). The majority of adolescents reported White/Caucasian as their ethnicity (78%). The second- and third- most reported ethnicity was East Asian (13%) and South Asian (10%), respectively, with remaining ethnicities as 2% Black, 1% Indigenous, 3% Middle Eastern, and 8% Biracial. Reflecting expected variability within a community sample, parents reported pre-existing mental health diagnoses for 17 adolescents: neurodevelopmental diagnoses (ADHD, ASD, LD) and/or mood and anxiety disorders.

### Procedure

Interested parents/adolescents were each emailed a Qualtrics link along with a unique code (if the adolescent did not have an email address, the parent was emailed both links) that allowed them to access the parent and adolescent questionnaire set, respectively, and complete forms. Data from this study were taken from a larger project examining parent and adolescent mentalizing, with parent and adolescent questionnaires each taking approximately 40 minutes. Only measures used for this study are reported here. Data storage adhered to institutional research ethics board requirements. As a token of appreciation, participants were entered into a draw to win one of three $50 gift cards. Additionally, if eligible, adolescents could put their participation hours towards high-school volunteer credits.

### Measures

#### Adolescent Difficulties

##### Strength and Difficulties Questionnaire

Adolescent difficulties were measured using the adolescent self-report Strength and Difficulties Questionnaire (adolescent-report SDQ) and parent-report SDQ. Both the adolescent-report and parent-report SDQ has been widely validated for measuring psychological adjustment and detecting emotional and behavioural problems (Bentley et al., 2019; Hill & Hughes, 2007; Van Roy et al., 2008; Wolpert et al., 2015). Adolescent internalizing symptoms were measured using the Emotional Problems subscale, and adolescent externalizing problems were measured using the Conduct Problems and Hyperactivity-Inattention subscales. Each subscale contains 5-items rated on a 3-point self-report scale from 0 (*not true*) to 2 (*certainly true*) for the period of the last six months. The adolescent-self report SDQ is framed in first person whereas the parent-report SDQ covers the same items but is framed in third person consideration of the adolescent. A score of adolescent difficulties was obtained by summing the items in each of these three subscales with a possible range from 0 to 30 (higher score reflecting greater difficulties). For this sample, the difficulty scores used for analyses had good internal consistency (adolescent-report, α = .76; parent-report, α = .81).

#### Household COVID-19 Disruption

COVID-19 disruption was assessed using the COVID-19 Family Stressor Scale (CoFaSS; Prime et al., 2021), a 16-item questionnaire filled out by the parent that assessed COVID-19 disruption within a household (i.e., “Since the start of the COVID-19 disruption, have any of the following changes occurred in your household?”) across three areas: Income Stress, Chaos, and Family Stress (Prime et al., 2021). The Income Stress subscale (5-items) measures parents’ experience of economic stress during the pandemic (e.g., “Applied for employment insurance or government assistance”), the Chaos subscale (4-items) measures experiences of chaotic states induced in response to the pandemic (e.g., crowded shopping areas, restricted supplies) and the Family Stress subscale (7-items) measures stressors stemming from family altercations and child management during the pandemic (e.g., “Inability to access educational materials for children”, “Felt crowded in my living space”). Parents rated the applicability of items since the onset of COVID-19 on a 3-point self-report scale from 1 (*not true*) to 3 (*very true*). The Total COVID-19 disruption scale, comprised of all items, measures the total level of COVID-19 disruption that parents experienced and has been demonstrated to have strong psychometric properties (Prime et al., 2021). Given our interest in parent-child dyads, we specifically examined the familial context using the Family-specific COVID-19 disruption subscale to measure disruptions within the family context. Within our sample, the internal consistency was strong for the Total score (α = .86) and the Family-specific subscale (α = .78).

#### Potential Moderating Parental Factors

##### Parental Reflective Functioning Questionnaire – Adolescent Version

PRF was assessed using parent reports on the Pre-mentalizing subscale of the Parental Reflective Functioning Questionnaire – Adolescent Version (PRFQ-A; Luyten et al., 2017b)^1^. While validation studies on the PRFQ-A are limited, use of the PRFQ-A with parents of adolescents has suggested good factor structure and reliability (e.g., Carlone et al., 2023; Szabó et al., 2022; Zayde et al., 2023). Further, the items are similar to the child-report version, which has strong psychometric properties and concurrent validity regardless of caregiver gender (Charpentier Mora et al., 2022; De Roo et al., 2019; Luyten et al., 2017a; Pazzagli et al., 2018; Rutherford et al., 2013). The pre-mentalizing subscale (6-items) measures an *inability* to understand underlying mental states that guide their child’s actions, feelings, and behaviours (e.g., pre-mentalizing subscale item “When my child is being difficult, he or she does that just to annoy me.”, Luyten et al., 2017b). Items are rated using a seven-point self-report scale from 1 (*strongly disagree*) to 7 (*strongly agree*). The Pre-mentalizing subscale score is the mean score of all scale items, with a possible range of 1–7. To aid in interpretation, the Pre-mentalizing subscale was reverse coded such that high scores indicate *strong* reflective functioning, and low scores indicate *weak* reflective functioning. For this sample, the internal consistency of the Pre-mentalizing subscale was .63 and was increased to .72 by removing one item (“I find it hard to actively participate in make believe play or imaginary activities with my child”), which was deemed to be age-inappropriate for the sample.

##### Depression Anxiety Stress Scales

Parental mental health was operationalized using the Depression subscale from the Depression Anxiety Stress Scales (DASS; Lovibond & Lovibond, 1995). Specifically, the 21-item short form (DASS-21) adapted from the original 42-item DASS. The Depression subscale (7-items) measures experience of dysphoric mood over the past week on a 4-point self-report scale from 0 (*did not apply to me at all)* to 3 (*applied to me very much, or most of the time*). The Depression subscale score is summed and multiplied by two with a possible range from 0 (no symptomology) to 42 (extremely severe symptomology). The Depression subscale has demonstrated good psychometric properties and reliability in non-clinical samples (Asghari et al., 2008; Lovibond & Lovibond, 1995; Sinclair et al., 2012). For this sample, the internal consistency of the Depression subscale was strong (*a* = .90).

##### Child and Adolescent Social Support Scale

Adolescents’ perception of parental support was measured with the Frequency portion of the parents subscale from the Child and Adolescent Social Support Scale (CASSS; Malecki et al., 1999). The Frequency subscale (12-items) measures how often adolescents received different types of support from their parents using a 6-point self-report scale from 1 (*never*) to 6 (*always*). The Frequency subscale score is summed from all items with a possible range from 12 (no experiences of support) to 72 (recurrent experiences of support). The Frequency subscale has demonstrated good psychometric properties with gender invariance (Demaray & Malecki, 2003; K. Malecki & Demaray, 2002; Rueger et al., 2008; Shinkawa et al., 2023). For this sample the internal consistency of the Frequency subscale was strong (*a* = .89).

#### General Parental Stress Measures

##### Schedule of Recent Experiences

Parents’ experience of stressful events was operationalized using the Schedule of Recent Experiences (SRE; Holmes & Rahe, 1967). The SRE is a 43-item checklist of positive and negative life events, that coincide with experience of psychological stress, over the last year. The item count score has a possible range between 0 and 43 (Pearson & Long, 1985) wherein high SRE scores indicate that an individual has experienced a large amount of stressful life events. The SRE has demonstrated good psychometric properties including predictive validity (Cleghorn & Streiner, 1979; Mendels & Weinstein, 1972).

##### Perceived Stress Scale

A general measure of parental stress was operationalized using Cohen’s Perceived Stress Scale (PSS; Cohen, 1994). The PSS (10-items) measures the perceived experience of stressful feelings or thoughts in the last month on a 5-point self-report scale 0 (*never*) to 4 (*very often*). A total score, representing the sum of items has a possible range between 0 and 40, with higher reflecting more unpredictable, uncontrollable, and overloaded participants feel their life has been in the past month (Cohen, 1994). The PSS has demonstrated good psychometric properties (Cohen, 1988; Mitchell et al., 2008; Taylor, 2015). Within our sample, the internal consistency of the PSS was strong (*a* = .88).

## Analysis Plan

Data were analyzed using SPSS 27.0 software and SPSS PROCESS macro program (Hayes, 2022). After addressing missing data, descriptive statistics were computed, and correlations were conducted to explore the associations among the key variables: PRF, parental depression, adolescents’ perception of parental support, COVID-19 disruption (parents’ report of Total COVID-19 disruption and parents’ report of Family-specific COVID-19 disruption), and adolescent difficulties (adolescent-reported difficulties and parent-reported adolescent difficulties)^2^. Second, regression models were utilized to explore whether the association between COVID-19 disruptions and adolescent-reported difficulties depends on the parental factors of interest. All predictor variables, including control variables (adolescent age and gender), the impact of COVID-19 disruptions, potential parental moderators (PRF, adolescents’ perception of parental support, and parents’ depressive symptoms) and their interactions with COVID-19 disruption, were mean-centered before analysis. Interaction terms were specifically included to explore how the combination of COVID-19 disruption and other moderators might affect adolescent outcomes. Initially, the regression incorporated total COVID-19 disruption as a predictor and used adolescent-reported difficulties as the dependent variable. Subsequently, to examine the effect of family-specific COVID-19 disruption, the model was rerun, replacing total COVID-19 disruption with Family-specific COVID-19 disruption. Third, the regression analyses were run again with parent-reported adolescent difficulties to explore whether similar patterns were found across informants. Lastly, a back-transformed average Fisher Z procedure was used to compare the strength of correlations between COVID-19 disruptions and adolescent difficulties with the correlations between other parental stress measures, such as major life events and perceived stress, and adolescent difficulties (these comparisons were done for both adolescent-reported difficulties and parent-reported adolescent difficulties). Data may be supplied on request of authors.

## Results

Missing data were analyzed separately for parent and youth data. Note, the measures obtained from adolescents directly were adolescents’ demographic data (i.e., age and gender), adolescent self-reported difficulties (YSDQ), and adolescents’ perception of parental support (CASSS); all other measures reported were completed by the parent. Three participants had incomplete datasets: One parent missed 2/25 items on the CoFASS total scale, while another parent missed 1/25 items on the CoFASSS scale (total missing parent data = .049%). One youth missed 12/24 responses on the CASSS scale (total missing youth data = 0.36%). Given the very small degree of missing data, missing data was handled using listwise deletion. Given the small number of participants with incomplete datasets (*n* = 1 youth, *n* = 2 parents), correlates of missingness were related to demographic characteristics, not reported in detail to protect participant anonymity.

At the subscale level, the data was inspected for outliers (i.e., +3*SD*), which were then winsorized to be within acceptable limits: adolescent-reported conduct problems (*n* = 1), parent-reported conduct problems (*n* = 1), total COVID-19 disruption (*n* = 1), PRF (*n* = 2), and life stressors total (*n* = 2). Prior to analyses, the distribution of variables were inspected with all falling within absolute values of skew <2 and kurtosis <7 (Ryu, 2011). The standardized residuals of all regressions were found to be within the Ryu (2011) criteria.

### Associations Between COVID-19 Disruption and Adolescent-Reported Difficulties

Correlation analyses (Table 1) revealed anticipated positive associations between the parents’ report of COVID-19 disruption (Total and Family-specific COVID-19 disruption) and the adolescents’ emotional and behavioural difficulties (adolescent-reported and parent-reported).^3^

**Table 1.** Pearson Correlations Among Variables of Interest.

Variable	*M*	*SD*	1	2	3	4	5	6	7	8	9	10
Adolescent Emotional and behavioural difficulties												
1. YSDQ	9.95	4.66										
2. PSDQ	7.10	4.98	.47***									
COVID-19 disruption measures												
3. CoFaSS total	23.87	6.05	.18	.32**								
4. CoFaSS family	10.54	3.11	.26**	.35***	.86***							
Potential parental moderating factors												
5. PRF	6.44	0.73	−.18	−.38***	−.02	−.15						
6. CASSS	40.46	10.06	−.40***	−.12	.03	−.06	.34**					
7. DASS	7.61	7.85	.08	.28**	.42***	.49***	−.15	.08				
Other parental stress measures												
8. SRE	6.45	4.06	.06	.08	.65***	.47***	.20	.19	.27**			
9. PSS	17.60	7.05	.07	.38***	.62***	.61***	−.06	.08	.57***	.41***		
Adolescent covariates												
10. Age	13.37	1.15	.13	.12	−.04	−.03	−.12	−.17	.00	−.06	−.11	
11. Gender	0.51	0.50	−.22*	−.10	−.02	−.02	.04	.01	−.06	−.03	−.13	−.01

*Note.* YSDQ and PSDQ refer to adolescent difficulties reported by the adolescent’s self-report (YSDQ) and parent’s report (PSDQ) of adolescent difficulties as reported on the SDQ. CoFaSS Total refers to parents’ report of total COVID-19 disruption as measured on the CoFaSS. CoFaSS Family refers to parents’ report of family-specific COVID-19 disruption, as measured on the CoFaSS Family subscale. PRF refers to reflective functioning as measured on the PRFQ-A Pre-mentalizing subscale (reverse scored). CASSS refers to adolescents’ perception of parental support as measured on the CASSS Frequency subscale. DASS refers to parental depression symptoms as measured on the DASS Depression subscale. SRE and PSS refer to life stressors and perceived stressors respectively, as reported by the parent. For gender, males were coded as 0 and females were coded as 1. For all continuous variables higher values reflect a greater endorsement of that construct.

*p < .05. **p < .01. ***p < .001.

#### Moderating Role of Parental Factors

Results of the regression analyses are provided in Table 2. The results are organized by the informant of adolescent difficulties and the specific COVID-19 disruption measure.

**Table 2.** Summary of Regression Models Predicting Adolescent Difficulties.

	Total disruption		Family disruption	
	Youth	Parent	Youth	Parent
Predictors	*B*(SE)/β	*B*(SE)/β	*B*(SE)/β	*B*(SE)/β
Adolescent age	0.34 (0.42)/0.08	0.61 (0.44)/0.14	0.30 (0.41)/0.07	0.69 (0.43)/0.16
Adolescent gender	−1.90* (0.90)/−0.20	−0.37 (0.95)/−0.04	−1.94* (0.90)/−0.21	−0.26 (0.95)/−0.03
CoFaSS	0.15 (0.09)/0.19	0.33** (0.10)/0.39	0.40* (0.17)/0.26	0.45* (0.18)/0.28
PRF	0.06 (0.67)/0.01	−2.43** (0.70)/−0.36	0.12 (0.67)/0.02	−1.78* (0.71)/−0.26
CASSS	−0.21*** (0.05)/−0.44	0.03 (0.05)/0.05	0.01 (0.05)/0.02	−0.19*** (0.05)/−0.41
DASS	0.01 (0.07)/0.02	0.07 (0.07)/0.10	−0.01 (0.07)/−0.01	0.09 (0.07)/0.13
CoFaSS X PRF	0.12 (0.15)	−0.38* (0.16)	0.20 (0.25)	−0.67* (0.27)
CoFaSS X CASSS	0.01 (0.01)	0.01 (0.01)	−0.01 (0.02)	−0.00 (0.02)
CoFaSS X DASS	−0.01 (0.01)	−0.01 (0.01)	−0.02 (0.02)	−0.01 (0.02)

*Note.* CoFaSS refers to parent’s report of COVID-19 disruption as measured on the CoFaSS with the specific subscale reported in the column headers. PRF refers to pre-mentalizing as measured on the PRFQ-A Pre-mentalizing subscale (reverse scored). CASSS refers to adolescents’ perception of parental support as measured on the CASSS Frequency subscale. DASS refers to parental depression symptoms as measured on the DASS Depression subscale. For gender, males were coded as 0 and females were coded as 1. For all continuous variables higher values reflect a greater endorsement of that construct. Parent indicates PSDQ and Youth indicates YSDQ.

*p < .05. **p < .01. ***p < .001.

#### Adolescent Report of Adolescent Difficulties

##### Total COVID-19 Disruption

The model including Total COVID-19 disruption was significant, *R*^*2*^ = .21*, F* (9, 78) = 3.64, *p* < .001. There was a main effect of adolescent gender and adolescents’ report of perceived parental support for adolescent-reported difficulties. Specifically, adolescents reported more difficulties when they were male and if they perceived less support from parents.

##### Family COVID-19 Disruption

The model that included family-specific COVID-19 disruption was significant, *R*^*2*^ = .22*, F* (9, 79) = 3.75, *p* < .001. Adolescent gender, family-specific COVID-19 disruption, and adolescents’ report of perceived parental support accounted for a significant amount of variance in adolescent-reported difficulties. Adolescents reported more difficulties when they were male as well as if they perceived their parents to provide less support. Moreover, adolescents reported more difficulties when their parents reported more family-specific COVID-19 disruption.

#### Parent Report of Adolescent Difficulties

To determine whether patterns of associations differed when the report of adolescent difficulties came from a parent (vs. the adolescents themselves), we conducted the same regressions noted above with the parents’ report of adolescent’s difficulties as the dependent measure (see Table 2). While there was a positive correlation between the informant reports (see Table 1), parents reported lower levels of adolescent difficulties as compared to adolescents, *t* (90) = 5.46, *p* < .001, with this effect found for each difficulty subscale, *p*s < .02.

##### Total COVID-19 Disruption

**Figure 1.**
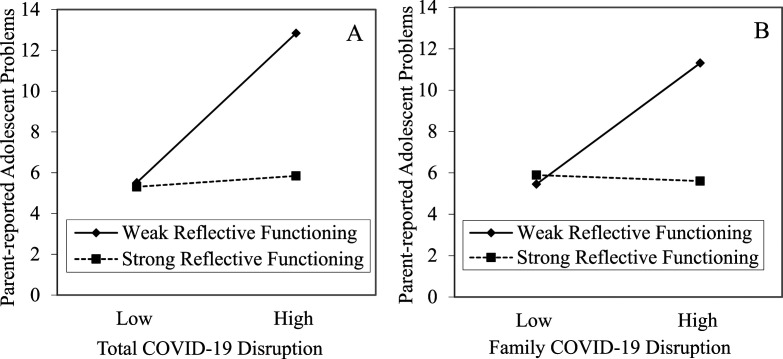
Parent-reported adolescent difficulties as a function of COVID-19 disruption and parental reflective functioning.

The model that included total COVID-19 disruption was significant, *R*^*2*^ = .25*, F* (9, 78) = 4.16, *p* < .001. Total COVID-19 disruption and PRF accounted for a significant amount of variance in parent-reported adolescent difficulties. Moreover, the interaction between total COVID-19 disruption and PRF was significant. The significant interaction suggests that the effect of total COVID-19 disruption on parent-reported adolescent difficulties depends on the parent’s level of PRF (see Figure 1(A)). Simple slopes for the association between total COVID-19 disruption and parent-reported adolescent difficulties were run at high and low levels of PRF, with low levels yielding a significant relation (*b* = 0.61, *p* < .001) and high levels yielding a nonsignificant relation (*b* = 0.05, *p* = .68). Thus, COVID-19 disruption is associated with more parent-reported adolescent difficulties when parents report weak reflective functioning skills, but not when parents report strong reflective functioning skills. In other words, strong PRF weakens the association between COVID-19 disruption and parent-reported adolescent difficulties.

##### Family COVID-19 Disruption

The model that included family-specific COVID-19 disruption was significant, *R*^*2*^ = .24*, F* (9, 79) = 4.08, *p* < .001. Family-specific COVID-19 disruption and PRF accounted for a significant variance in parent-reported adolescent difficulties. Moreover, the interaction between family-specific COVID-19 disruption and PRF was significant, suggesting that during the pandemic the association between disrupted family functioning and parent-reported adolescent difficulties depends on the parent’s level of PRF (see Figure 1(B)). Simple slopes for the association between family-specific COVID-19 disruption and parent-reported adolescent difficulties were run at high and low levels of PRF, with low levels yielding a significant relation (*b* = 0.95, *p* < .001) and high levels yielding a nonsignificant relation (*b* = −0.04, *p* = .87). Thus, family-specific disruptions during the pandemic are associated with more parent-reported adolescent difficulties when parents reported weak reflective functioning skills, but not when parents reported strong reflective functioning skills.

### Associations Between General Parental Stress Measures and Adolescent Difficulties

To examine the relationships between COVID-19 disruption and adolescent difficulties compared to other measures of parental stress, we analyzed correlations among life events, perceived stress, and adolescent difficulties (both adolescent and parent reports; see Table 1). We then compared the strength of these correlations to those with COVID-19 disruption using a back transformed average Fisher Z procedure for comparing overlapping correlations (Hittner et al., 2003). Of the eight comparisons, three were significant, and one marginally significant (all other comparisons, *p*s > .14), and majority reflected stronger associations between COVID-19 disruption with adolescent difficulties than other stress measures^4^. The comparisons revealed stronger associations between family-specific COVID-19 disruption with adolescent-reported difficulties as compared to perceived stress, z = 2.05, *p* = .04 and life stressors, z = 1.90, *p* = .06, as well as with parent-reported adolescent difficulties as compared to life stressors, z = 2.53, *p* = .01. Furthermore, the comparisons revealed stronger associations between total COVID-19 disruption with parent-reported adolescent difficulties compared to life stressors, z = 2.87, *p* = .004. Thus, there is some evidence that COVID-19 disruption more strongly relates to adolescent difficulties than other measures of parental stress; however, this was not consistently found.

## Discussion

Numerous studies have documented the negative impacts of COVID-19-related disruptions on youth mental health (De France et al., 2022; Ma et al., 2021; Westrupp et al., 2021). This study aimed to explore whether specific parental factors moderated the strength of the association between COVID-19 disruption (for a household) and adolescent difficulties. We found that adolescents’ report of perceived parental support and PRF were key factors relating to adolescent difficulties: perceived parental support uniquely related to adolescent-reported difficulties; PRF moderated the association between COVID-19 disruption (total and family-specific) and adolescent difficulties (albeit only for parent-report). The findings have implications for supporting families experiencing unpredictable circumstances. Moreover, as adolescents reported greater difficulties than the parent reports, findings highlight the value of considering both adolescent and parent perspectives.

### The Association Between COVID-19 Disruption and Adolescent Difficulties

Building off previous research (Donker et al., 2021; Low & Mounts, 2022; Operto et al., 2022; Peltz et al., 2023; Romero et al., 2020; Shelleby et al., 2022; Westrupp et al., 2021) documenting the negative effects of COVID-19 disruption on adolescent mental health, we hypothesized there would be a positive association between COVID-19 disruption and adolescent difficulties. Our results are aligned with this previous research: At the bivariate level, household COVID-19 disruption was weakly positively related with both adolescent-reported and parent-reported adolescent difficulties. In general, this pattern of results held when controlling for adolescent gender, adolescent age, and parental factors (PRF, parental depression, and adolescent-reported parental support). Total COVID-19 disruption and family-specific COVID-19 disruption were positively associated with both adolescent-reported and parent-reported adolescent difficulties with one exception: Total COVID-19 disruption was not associated with adolescent-reported difficulties when parental factors were included (though family-specific disruption was still significant). As we cannot determine the direction of association in this study, it may be that COVID-19 disruptions had a negative impact on adolescents, manifesting in more emotional and behavioural difficulties in both adolescent and parent reports (De France et al., 2022; Ma et al., 2021; Westrupp et al., 2021). Alternatively, it may be that for those families whose adolescents have more difficulties (as reported by both the adolescent and the parent), COVID-19 disruptions within a household are experienced more greatly. The heightened disruptions experienced by these families may be due to reduced access to mental health services during the pandemic, making it difficult for adolescents with significant needs to access necessary care. Indeed, the pandemic disrupted the continuity of care, led to an increased demand for mental health services, and worsened care inequalities, which could have posed significant barriers for adolescents seeking help (Stepanova et al., 2024).

### Parental Factors

Central to our aim, we examined whether specific parental factors (PRF, parental depression, and perceived parental support) moderated the strength of this association or operate as a unique predictor of adolescent difficulties.

### Parental Reflective Functioning

We hypothesized that associations between PRF and adolescent mental health would replicate those observed with children (Slade, 2005; Smaling et al., 2016; Steele et al., 1999) in that PRF would be associated with fewer adolescent difficulties (through the occurrence of more positive parent-adolescent interactions among parents with better PRF). When controlling for adolescent gender, adolescent age, COVID-19 disruption, and other parental factors (i.e., parental depression and adolescent-reported parental support), PRF was not significantly associated with adolescent-reported difficulties but was weakly negatively associated with parent-reported adolescent difficulties. Moreover, we expected stronger PRF would weaken the association between COVID-19 disruption and adolescent difficulties. Support for this hypothesis was found: COVID-19 disruption was associated with more parent-reported adolescent difficulties when parents reported weak reflective functioning skills (i.e., high pre-mentalizing), but not when parents reported strong reflective functioning skills (i.e., low pre-mentalizing). Thus, as predicted, strong reflective functioning skills appear to weaken the association between COVID-19 disruption and parent-reported adolescent difficulties. These findings are consistent with previous research that found that maternal mentalizing moderated the association between mothers’ anxiety – which was increased during COVID-19 – and children’s (3- to 6- years old) externalizing problems (Dollberg et al., 2021).

One potential explanation for our findings is that during times of high COVID-19 disruption, parents with strong PRF skills are more attuned and responsive to the needs of their adolescent, who may too be experiencing the disruptions; while parents with weak PRF skills may be less able to address their adolescent’s needs, with this lowered sensitivity elevating parent-reported adolescent difficulties. This explanation would be in line with research demonstrating a significant association between weak PRF, specifically parent pre-mentalizing, and reduced communication and involvement with the child, as reported by the parent (Rostad & Whitaker, 2016). It may also be that parents with strong reflective functioning skills are better able to recognize their own internal state and behaviour in relation to their child, and thereby manage their own reactions to COVID-19 disruptions in a way that reduces potential negative impacts on their adolescents leading to more stable parent-reported adolescent difficulties, as compared to parents with weak reflective functioning skills. Liu and Doan (2020) describe a “spillover effect” to occur when “the exposure or experience of stress in one domain influences one’s ability to function optimally in another domain” (Liu & Doan, 2020, p. 850). As weaker reflective functioning is associated with lower distress tolerance (Rutherford et al., 2015), parents with weak reflective functioning skills may be less able to tolerate high levels of household COVID-19 disruption, which reduces their ability to attend to their adolescent’s needs.

It is important to note that PRF did not moderate the association between COVID-19 disruption and adolescent difficulties when examining adolescents’ report. Situational specificity may contribute to the divergence between the effects we observed with parent-reported and adolescent-reported difficulties. Parents’ reports of adolescent difficulties may be based on what they can observe in their adolescent’s behaviour during parent-child interactions or contexts where they are present (e.g., within the home). Since parents are aware of the difficulties their adolescents experience in these contexts, their ability to interpret and respond to their adolescent’s needs, facilitated by PRF, may play a more significant role. In contrast, adolescents’ self-reports likely capture experiences within a broad range of contexts, including those outside the parents’ awareness or involvement, such as school or peer relationships. Thus, PRF may play less of a role. Regardless of interpretation, the differential patterns underscore the importance of considering situational specificity in understanding discrepancies between parent and adolescent reports.

### Parental Depression

Parental depression was positively correlated with parent-reported adolescent difficulties, though this did not hold when adolescent age/gender and other parental factors were included within a regression model. Moreover, we did not find support for our hypothesis that parental depression would strengthen the association between COVID-19 disruption and adolescent difficulties for either adolescent reported or parent-reported adolescent difficulties (as per past work showing parental depression to negatively affect the parent-child relationship resulting in increased reports of adolescent difficulties; Shafer et al., 2017). While our results suggest that parental depression does not play a moderating role in the association between COVID-19 disruption and adolescent difficulties (both adolescent-reported and parent-reported), it is important to note possible methodological considerations for our findings. In particular, in our sample, levels of parental depression were low, with the majority (81%) of parents scoring within the normal and mild range (0–13) indicting that any symptoms present are generally not severe enough to interfere significantly with daily functioning; only a small subset (10%) of parents scoring within the severe range (21 or greater) indicating that their symptoms negatively affect their ability to function in various areas of life. This limited variance in parental depression could have constrained the sensitivity needed to detect moderating effects of depression on associations between COVID-19 and adolescent difficulties.

### Perceived Parental Support

Parental support has been identified as a protective factor for adolescent emotional and behavioural outcomes (Barnes et al., 2006; Gorostiaga et al., 2019; Sheeber et al., 1997; Wang et al., 2013). Consistent with this past work, as adolescent-reported parental support increased, there was a decrease in adolescent-reported difficulties – even when controlling for the influence of COVID-19 disruption, PRF, and parental depression. This finding is consistent with findings from McArthur and colleagues (2023) who, using a pre-post pandemic design, found that children’s report of a sense of connectedness to caregivers was associated with lower COVID-19 anxiety and depression symptoms in children aged 9- to 11- years old (McArthur et al., 2023). Interestingly, while greater perceived parental support was correlated with PRF (suggesting some consistency with how adolescents and parents view parenting styles), we did not find associations between adolescents’ report of perceived parental support and parents’ report of adolescent difficulties. As well, contrary to predictions, adolescent perceptions of parent support did not modulate the association between COVID-19 disruption (neither total COVID-19 disruption or family-specific COVID-19 disruption) and adolescent difficulties, regardless of the source of the report. Thus, while increased parental support (or adolescents’ perceptions thereof) may reduce adolescent-reported difficulties (or adolescents with fewer difficulties are more likely to perceive parental support), it seems to operate independently from (vs. interacting with) external stressors like COVID-19 disruption. Nonetheless, with PRF, these findings offer support for the importance of parenting behaviour for adolescent well-being.

### Comparisons with Other Parental Stress Measures

As there are few studies investigating how COVID-19 disruption (reflecting a global, shared environmental context) is similar/distinct from other parental stress, we investigated whether associations between COVID-19 disruption with adolescent difficulties would differ with non-covid-specific measures of stress, including major life stressors and perceived stress. By doing so, we hoped to be able to provide information as to how the proliferation of work on COVID-19 disruption may provide insight into other stressful contexts. However, we acknowledge that within our study, the other stress measures were completed by parents during COVID-19, meaning they are not independent from the pandemic context.

As COVID-19 was a novel stressor for which the world was not prepared, we anticipated that the disruption felt by parents would show greater associations with adolescent well-being than other measures of parental stress. Supporting this notion, we observed several instances where the correlations with COVID-19 disruption and adolescent difficulties (across both adolescent and parent-report) were stronger than those for life stressors or perceived stress. These findings suggest that there are instances when COVID-19 disruption is associated with more variance in adolescent difficulties (both adolescent-reported and parent-reported) than does a parent’s perception of stress or a parent’s experience of general life stressors. That said, the directionality underlying this association cannot be assumed, it may be that if adolescent difficulties are higher (as reported by either the adolescent or the parent), a parent’s experience of COVID-19 household disruption, relative to their experience of other stress, is greater. Despite our measurement confounds, these findings are consistent with previous work showing that the associations between pandemic-related stress and greater child emotional and behavioural problems are unique as they could not be explained by parent’s general stress levels (Whittle et al., 2020). One potential explanation for our findings is that parents’ report of COVID-19 disruption is not unique to them as parents, but reflects the general impact on the household (thus capturing disruption for adolescent themselves as well). COVID-19 provided an example of how a wide-reaching disruption may affect parents, families, and adolescents; these nuanced relationships may also be important for understanding and anticipating the effects of other forms of environmental and societal disruption families may encounter in the future (e.g., endemics/pandemics, natural disasters, conflict).

It is also important to note that we did not consistently find differences in the strength of associations. As mentioned, the measurement confound of collecting the general stress measures during the pandemic context meant that our general stress measures are not independent from the pandemic context. Specifically, greater levels of pandemic disruption likely confounded how respondents perceived unrelated stressors, and increased likelihood of stress events (e.g., loss of job). Future work could examine whether similar patterns emerge during non-pandemic times.

### Limitations and Future Research

The current study has several limitations that may impact the generalizability of our findings. Most critically, it should be reiterated that our cross-sectional study provides a snapshot of the disruption experienced by families due to the COVID-19 pandemic, and how it related to parental factors (PRF and parental depression), adolescents perception of support, and adolescent difficulties. This does not allow us to infer causal direction and longitudinal work is required to investigate mechanistic and potential sleeper effects. Expanding on the aspect of time, data collection begun in April 2021 and ended in March 2022 capturing the third wave of rising COVID-19 cases in Canada and corresponding restrictive public health measures. As such, the panic and novelty associated to the COVID-19 pandemic was likely reduced in our sample as individuals adapted. While our findings represent parent’s experience during the pandemic, they may not be representative of early onset (and potentially more acute) experiences.

Another important consideration is that the majority of our significant effects were found within-informant models, where both the independent and dependent variables were reported by the same individual. While differential patterns may reflect situational specificity, it also raises the possibility of shared method variance influencing our findings. As such, it is important for future research to replicate findings with further use of cross-informant models.

Our findings should be considered in the context of our sample and replicated before firm conclusions can be made. Future research should seek to confirm the observed findings in a larger, more diverse sample. In particular, with a larger sample it would be useful to examine further moderation effects, for example whether the interaction between household disruption and PRF differs by adolescent gender. As conceptualizations and value of PRF may differ cross-culturally (Lee et al., 2023; Stuhrmann et al., 2022; Sümer et al., 2024), the findings need to be considered within the sample used, namely, majority White with higher education. As well, the majority of parents were mothers, which is a noted limitation in the PRF literature, generally (e.g., Camoirano, 2017; Dinzinger et al., 2023). Finally, restricted range in these measures (i.e., generally strong PRF, normal to mild severity of parental depression) may have attenuated the strength of our findings.

## Conclusion

While the COVID-19 pandemic is (mostly) behind us, understanding the association between the disruption it caused and adolescent emotional and behavioural difficulties, and the role parental factors may have on this association is crucial in informing how best to support families as they navigate unpredictable circumstances. The association between the level of COVID-19 disruption experienced within a household and adolescent difficulties (regardless of the source of report) may reflect that for families whose adolescents had pre-existing mental health challenges the experience of COVID-19 disruptions within a household was heightened. The increase of COVID-19 disruptions experienced by families with adolescents facing more difficulties may be facilitated by reduced access to necessary services during the pandemic. As such, it is important to recognize that during societal disruptions, such as the pandemic, negative experiences may be exacerbated for families already facing challenges. Thus, during times of disruption, it is essential to prioritize continuity of care and ensure that access to mental health services remains available. Our findings highlight that while the level of COVID-19 disruption within a household related to adolescent difficulties (both adolescent-reported and parent-reported), this relationship varied based on parental factors (specifically PRF on parent-reported difficulties). In addition to theoretical implications (e.g., family systems theory, role of PRF within adolescence), findings have practical implications. For parent reports, we found that PRF may serve as a protective factor, wherein the association between COVID-19 disruption and adolescent difficulties was attenuated when parents reported better abilities in detecting and understanding their children’s internal mental states. Thus, during times of disruption (or within populations where household disruption is high), it may be advantageous to implement programming to increase parents’ sensitivity to the mental states of their children. For adolescent reports, perception of parental support was generally associated with fewer adolescent difficulties, rather than affecting COVID-19 disruption associations with difficulties. Thus, the promotion of universal parenting programs and/or general policies to enable parents with increased flexibility to provide support (e.g., flexible hours or remote work options) may provide benefit for adolescents’ well-being. Moreover, findings have methodological implications with discrepancies in the results indicating that adolescents and parents may use different cues to assess difficulties, with these varying perspectives offering different insights into possible ways to support adolescents during stressful contexts.

## Notes

1 The PRFQ-A is comprised of three subscales: Pre-mentalizing, Certainty of Mental States, and Interest and Curiosity in Mental States (Luyten et al., 2017b). Items from the Certainty of Mental States subscale and Interest and Curiosity in Mental States subscale were not included in the analyses due to marked discrepancies in the literature as to how to score and interpret these subscales (e.g., Anis et al., 2020; Luyten et al., 2017a; Pajulo et al., 2018).

2 To assess differences in COVID-19 disruption over time, participants were grouped by their participation periods: April-June 2021, July-September 2021, October-December 2021, and January-March 2022. Parents who participated in April-June 2021 and October-December 2021 reported significantly more disruption on the CoFASS total score than those who participated later (April-June 2021 vs. January-March 2022: *p* = .02; October-December 2021 vs. January-March 2022: *p* = .04). This may reflect changes in policies throughout the pandemic, particularly regarding online versus in-person schooling (e.g., in Ontario, where most participants were recruited, children experienced phases of returning to in-person schooling, interrupted by temporary online learning, with a permanent return in April 2021). Our primary focus was the association between COVID-19 disruption and youth difficulties, and since additional analyses showed no significant differences in the strength of this association based on participation timing (*p*s > .25), timing was not included in further analyses.

3 One participant who identified as genderfluid was excluded from analyses that involved gender due to the insufficient number of participants comprising this category for meaningful interpretation.

4 The association between perceived stress and parent-reported adolescent difficulties was stronger than the associations between COVID-19 disruption and parent-reported adolescent difficulties. However, these differences were not significant (*ps* > .54).
